# Can people with longstanding bulimia nervosa suffer from severe and enduring eating disorder? A qualitative study

**DOI:** 10.1186/s40337-024-01161-2

**Published:** 2024-12-18

**Authors:** Paul H Robinson, Giulia Guidetti, Jessica Kasriel, Jomana Khawandanah, Maxine Hughes, Zeinab Hachem

**Affiliations:** Division of Medicine, University College London, London, UK

**Keywords:** Bulimia nervosa, Severe and enduring, Eating disorder, Qualitative, Thematic analysis

## Abstract

**Objectives:**

To interview a series of individuals with bulimia nervosa of longstanding to establish their symptoms and examine the suggestion, using qualitative analysis, that the term “Severe and Enduring Bulimia Nervosa (SEED-BN)” might be appropriate and helpful.

**Methods:**

12 participants with Bulimia Nervosa, one male, were interviewed with the help of an interview guide. Interviews were recorded and transcribed. Transcripts were analysed using Thematic Analysis.

**Results:**

Participants with Bulimia Nervosa described serious problems in many realms, especially social, psychological, family and relationships.

**Conclusions:**

Bulimia nervosa of long duration is associated with many serious symptoms. It seems likely that recognition of long-standing bulimia nervosa as a severe and enduring eating disorder could encourage clinicians and families to pay attention to the wide variety of problems suffered by this group. Further research is required to examine this proposal.

## Abstract (highlights)

People can suffer from eating disorders (EDs) for many years. The term Severe and Enduring Eating Disorders (SEED) has been used in order to draw attention to the many difficulties, physical, psychological, social and family, suffered by people living with longstanding EDs. The concept of SEED has been applied to Anorexia Nervosa and the term Severe and Enduring Anorexia Nervosa (SE-AN) has been coined to refer to them. We have seen many people with long term Bulimia Nervosa (BN) with binge eating, vomiting, abuse of laxatives and body image problems, and believe that they too can be thought of as suffering a severe and enduring illness (SEED-BN or SE-BN) requiring attention to a wide variety of difficulties. We interviewed 12 individuals with BN of at least 5 years duration using qualitative research interviews and analysed the interviews to identify themes emerging. We confirmed that people living with longstanding BN have serious problems in a number of areas including social life, relationships, mental health and physical health. Although we believe that further studies are required, the term SEED-BN or SE-BN can reasonably be used in the group of people with BN of long duration who have significantly reduced quality of life.

## Background

Bulimia nervosa (BN) gives rise to substantial psychosocial morbidity. Studies examining disability have determined that BN is associated with 33.8 years lived with disability (YLD) per 100,000 people, compared to 9.4 YLD for Anorexia Nervosa [26]. Treatment of BN and BED (Binge Eating Disorder) has advanced considerably but meta-analysis has suggested remission rates of only around 30% by the end of treatment for BN [15]. Hence, many sufferers from these eating disorders continue to have symptoms and they may require different approaches from those they have already received [1, 14].

People with intractable eating disorders, termed Severe and Enduring Eating Disorder (SEED) [18–20] find themselves faced with enduring long-term symptoms which are associated with substantial distress and morbidity. Most studies on SEED have focussed on Anorexia Nervosa (AN). However, a few have included Bulimia Nervosa (BN). Piñar-Gutierrez et al. [16] demonstrated high levels of psychiatric and physical morbidity in 67 individuals with longstanding BN in Spain, Moreover, Lindgren et al. [13] in a Swedish qualitative study of 14 women with BN with variable lengths of illness, identified “feeling stuck” and feeling identified with the illness in a study of young women attempting to recover from BN. Grogan et al [11], in an Irish qualitative study of a group of 15 people with a variety of eating disorders including bulimia nervosa and 15 clinicians, identified personal resilience as an important factor proposed to precede recovery from long term eating disorder. Resilience was also identified by Las Hayas et al. [12] as a significant aspect of improvement in eating disorders recovery in a qualitative study in Spain of a variety of eating disorders in 20 women, 6 of whom had a diagnosis of BN, also studying 8 experts in eating disorders and 6 caregivers. In a qualitative study of relapse in BN, Wasson [27] identified internal states and relationships as the major triggers for relapse in 26 US women with bulimia nervosa who were recruited from Overeaters Anonymous. Treasure et al [24], examining the concept of SEED, concluded that it had relevance for both AN and BN. In summary, in the few studies in which BN has been a focus of attention, a chronic course of the illness has been identified and predisposing factors in both relapse and recovery have been identified.

This study aimed to be an in-depth exploration of the lived experience of longstanding BN using qualitative methodology to investigate in 12 individuals with longstanding BN a wide range of experiences ranging from physical and psychological symptoms to impacts on social and family life, occupation and finances.

## Methods

### Participants

Adult participants with BN, 12 in total, were recruited originally from the outpatient department of a large Eating Disorder Service in London between 2014 and 2016. Demographic information is provided in Table 1. Participants were included if they were over 18 and had a clinical diagnosis by the senior author (PR), using DSM-5 criteria, of BN for at least 5 years continuously. The number of participants was estimated based on the advice of Guest et al. [10] that for most qualitative studies, 12 was a reasonable number of participants. We therefore approached 12 participants and all agreed. Each participant was interviewed after reading an information sheet, then signing a hard copy consent form at least 24 h later, having had that time to ask questions. Interviews took place in a quiet room in the clinical unit and each was audio recorded and then transcribed. Recordings were labelled with a research ID unrelated to the participants’ names or initials and kept on a secure folder on the Hospital Trust computer system and securely deleted as soon as the transcripts had been checked for accuracy, within 10 days of recording. Only the interviewer and the senior research (PHR) had access to the recordings. In this report participants’ initials have been changed to preserve anonymity.

**Table 1:** Demographic information on 12 participants with longstanding Bulimia Nervosa

**Age**	26–58	Mean 35.6		
**Ethnicity**	UK White 7	Black/Mixed 2	East Asian/mixed 2	Other 1
**Gender**	Female 11	Male 1	F 92%	
**Length of history (years)**	6–29	Mean 15.7		
**Living arrangement**	Alone 7	With partner 1	With family 2	Flat/house share 2
**Substance misuse**	Yes 3	No 9	Yes 25%	

### Interviews

Interviewers were all the co-authors apart from PHR who convened the other co-authors in qualitative discussion groups. Each of the 5 interviewers saw 2 or 3 of the participants. All interviewers were Masters students at University College London and held an honorary contract with the mental health trust where the participants were being treated. They were not involved in treatment of the participants. The interviewers interviewed the participants with the help of a topic guide (see Table 2) with questions that covered areas around physical, psychological, family, occupation, accommodation, finances, relationships, and social realms. The topic guide was developed from content in “Severe and Enduring Eating Disorders” [18]. However, the topic guide was used flexibly and interviewers were free to explore aspects of participants’ lives as they felt was appropriate according to the issues that the participant was sharing.

**Table 2:** Starting questions in each realm in the research interviews

Realm	Sample initial question
Social	Can you tell me how the eating disorder has impacted your social life?
Relationships	Can you tell me how the eating disorder has impacted your relationships?
Family	How has the eating disorder affected your family life?
Psychological	How has the eating disorder affected your mood and feelings?
Finance	Have you had any financial strains as a result of the eating disorder?
Occupation	How has the eating disorder affected your experience at work/education?
Physical health	What effects have the eating disorder had on your physical health?
Treatment	Tell me about your experience of treatment for your eating disorder

The questions were kept open-ended with minimal interruption by the interviewer. Participants were asked to describe their experiences and interviewers responded by asking further open questions until the matter being discussed had been as fully described as possible. The duration of the interviews ranged between forty minutes and two hours. Interviews were recorded and transcribed.

### Analysis

Each of the interview transcripts was analysed using thematic analysis [4]. The specific form of TA was Reflexive Thematic Analysis. “Reflexive thematic analysis is considered a reflection of the researcher’s interpretive analysis of the data conducted at the intersection of: (1) the dataset,(2) the theoretical assumptions of the analysis, and; (3) the analytical skills/resources of the researcher [3].” (From Byrne [7]. This approach was chosen as we required a flexible approach, not tied to a specific theoretical framework, that could be extended to larger sample sizes.

Braun and Clarke [2, 4] list the characteristics of this approach as:

1. Dataset familiarisation
2. Rigorous and systematic coding
3. Exploring, developing and refining themes
4. Producing the analytic report

While the realms were predetermined, came from the researchers’ experience of treatment of eating disorders, and therefore followed a deductive paradigm, coding, themes and interpretation were all carried out inductively without prior assumptions. The researchers also used some elements of phenomenology in that the subjective experience of participants was explored.

**Fig. 1:**
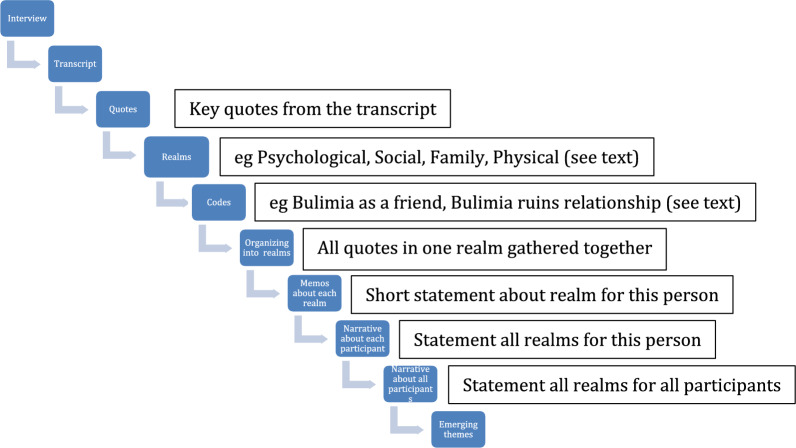
Illustrating the process used to analyse interview transcripts in the study

Quotes from the interviews were transferred to an Excel spreadsheet and given initial open codes that briefly described the quote. These initial open codes were further classified into selective codes that serve the purpose of capturing common patterns within the original realms of the interview guide. The selective codes were then grouped together under each realm, and a descriptive memo was created for each of the realms. A general narrative was then written about each participant which included their general experience, emerging themes and the researcher’s interpretation. Common patterns and themes were then compared between different participants in order for conclusions to be made between groups. Codes, memos, themes and narratives were discussed regularly in a qualitative analysis meeting with at least the interviewer/analyst and the senior researcher (PHR). The process is illustrated in Fig. 1.

### Relationship between the contributions of the individual researcher and the group

Each researcher analysed the data obtained in the interviews and their transcripts drawing on their own theoretical assumptions and their own particular skills (see above quote from Byrne [7]. At the QA meeting noted above, researchers and the senior researcher (PHR) met to discuss the quotes, codes and themes and came to agreement on each analysis going forward.

## Findings

### Participants

Of the 12 participants the mean age was 35.6 years and the mean length of illness was 15.7 years, with a range of 6 to 29 years of illness. There was only one male in the group. 7 were of UK white ethnicity, 2 black, 2 mixed East Asian and one Latina.

### Themes emerging

The themes that emerged from the qualitative analytic process are listed in Table 3, under the predetermined realms.

**Table 3:** Themes emerging within each realm

Realm	Themes
Social	Distressing isolation
	Welcome isolation
	Solitary living
	Eating disorder keeps me company
Relationships	Intimacy avoided
	Fear of exposing body
	Sex as a distraction
	Male partners shape obsessed
	Helpful structure
	Presence of partner reduced symptoms
Family	Pressure to be thinner
	Dismissing and trivialising the Eating Disorder
	Children aggressive
	Children developing Eating Disorders
	Children helpful when present in reducing symptoms
Psychological	Eating calms me
	Eating Disorder as a friend
Finance	BN leads to shortage of money
Occupation	Only works when feels thin
	BN distracting at work
Physical health	Dental problems
Treatment	Therapy sometimes helpful
	Losing faith in therapy

1. Social life
  Themes: Distressing isolation, Welcome isolation, Solitary living
  Almost all participants described how the eating disorder profoundly affected their social lives, rendering them isolated. Nine out of the twelve participants with BN lived either alone or shared with strangers. For some the eating disorder caused severe, distressing isolation, which sometimes provoked episodes of bulimia while for others that isolation was welcome, as it allowed the person to engage in eating disorder behaviours without interruption. Bulimia for some participants became a priority activity and social engagements were avoided or cancelled in order to facilitate bulimia.
  Quotes:
  *I do feel quite lonely at the moment which is triggering more bingeing definitely*
  Solitude was also valued:
  *I live on my own in this flat and I like it. I'm used to it now, I don't know if I would love to have someone living with me, because I do whatever I want whenever I want at the moment.*
  While for some it had both negative and positive aspects:
  *It's like a cycle, you isolate yourself in order to be in the act of binge and purge and it becomes the reason for isolating yourself and it's a reason and an excuse and it also perpetuate the isolation*
  And bulimia could act as a “friend”:
  *But when I isolate, I don't feel isolated because I've got this kind of friend (bulimia) with me, keeping me company.”*
2. Relationships
  Romantic relationships were avoided by two thirds of our participants. (Theme: Intimacy avoided). Fear of intimacy was described, fear of exposing the body in sexual relationships (Theme: Fear of exposing body), feeling too fat for sex but sometimes enjoying sex as a way to keep bulimia at bay (Theme: Sex as a distraction), and feeling too vulnerable for a relationship were all described. Several participants described their male partners as “shape obsessed” (Theme) and this made their bulimia harder to control.
  Quote:
  *I keep everyone at arm’s length, as I want to keep my vulnerabilities concealed. it’s a lot for someone to take. I find relationships too intense to manage, they’re always breaking up. If I had a boyfriend, I would have to keep him in the freezer and defrost him when I was in the mood to chat to him*
  A relationship was in fact sometimes very helpful (Themes: Helpful structure, Presence of partner reduced symptoms):
  *Quote: Having him there gave a lot more structure to the day, so actually it really helped, I was cooking dinner for 2 people and we’d eat dinner together*
3. Family
  Participants reported both positive and negative experiences with their families, although there were more negative than positive comments. In some cases there was substantial pressure to lose weight (Theme: Pressure to be thinner) and one participant’s mother had an eating disorder which the participant felt had affected her (the participant) adversely. Dismissing and trivialising the condition (Theme) was experienced by several participants and families were often described as absent or lacking warmth and support. Children were sometimes aggressive (Theme) with their eating disordered parent in dealing with the symptoms and some seemed to be affected by the eating disorder, developing the conditions themselves (Theme: Children developing Eating Disorders). On the positive sided, some of the women found their partners helpful and their bulimia reduced when the partner was around (Theme: Presence of partner reduced symptoms), and one found that she purged less if her children were around. (Theme: Children helpful when present in reducing symptoms.)
  Quotes:
  *In my family we find it hard to express emotions. They are all completely dishonest and there’s no emotional closeness: We were just brought up in this environment where we can’t tell anyone what we really think…you can’t have one cross word with them. Because it’s just too fragile and that’s just not what we do.*
  For participants with children it was hard to fit bulimia into the busy day:
  *After the children have gone to bed I would binge and it would be bread, cheese and pasta and when I purge then I would have some more and I would get to the point to throw everything up in the space of 2-3 hours.*
  One participant removed the toilet door so her children would see her vomiting, which reduced her vomiting in that toilet. However, she took a large plastic bin to her room into which she used to vomit.
4. Psychological issues
  The participants described many emotional difficulties including loneliness, feeling vulnerable, feeling “mad”, feeling fat when at normal weight and abusing alcohol to facilitate binge eating and to deal with low mood and despair. Some women described bulimia as a “friend” which mitigated isolation. (Theme: Eating Disorder as a friend).
  Quotes:
  *If I have a problem, I start eating food, it calms me down.(Theme:* Eating calms me)* I was bullied because of being overweight as a child and that made me start bingeing as a teenager*
  For some there was a link with alcohol:
  *If I’ve had a good day and I then felt that I’ve been really in control and then I go out and drink, well I lose more control*
5. Finances
  For many, bulimia and binge-eating was a costly behaviour. (Theme: BN leads to shortage of money) Participants described financial difficulties due to the expenses of food, and one could not afford to have her teeth, crumbling due to vomiting, treated. Most spent over £20 on binge foods, on days they did binge, which could be every day.
  Quotes:
  *I don’t have savings and I don’t go wild each month. Yes I do it on food or whatever but I don’t buy nice clothes, I don’t go on holidays, I don’t splurge because I’m never in a position to.*
6. Occupation
  Half the participants with bulimia nervosa had full or part time jobs. Some participants with bulimia nervosa would not go to work unless at a certain (thin) size. (Theme: Only works when feels thin). Those at work found that the eating disorder distracted from tasks at their jobs.
  Quotes:
  *My work schedule is very flexible, and I can chose to work from home if I wish. I enjoy this flexibility but find that working from home often results in binges and purges, and I think that more structure would improve my eating habits.*
7. Physical health
  The participants with bulimia nervosa had dental problems and various gastrointestinal symptoms, but most did not complain of serious physical symptoms.
  Quote:
  *I can't even bite an apple and cut my food. My teeth are ruined and eroded because of vomiting. (Theme:* Dental problems) *I feel tired all the time. I take sleeping pills before I go to bed. I feel tired all the time. It affected my bones, my hips, my teeth, and my heart.*
8. Treatment
  Most of the participants had received therapy and although one commented that it was helpful to talk in a group (Theme: Therapy sometimes helpful), little benefit had accrued. Several had lost faith (Theme: Losing faith in therapy) in conventional treatment and were seeking alternative approaches. Poor attendance at therapy was common.
  Quotes:
  *I first had treatment when I was 17 and had had BN for about a year. In the first therapy I hated it and did not like the therapist so I only attended a few sessions. Then at 18 I was referred to elsewhere and I attended CBT sessions for 6 months but found it unhelpful and stopped going in favour of going to uni. In my 2nd/3rd year at uni I went for treatment again and attended group therapy but again I didn't like this so only attended 4 or 5 sessions.”*

### Trustworthiness of the data

The assessments suggested by Connelly (2016) [8] were applied as follows:

*Credibility* The study was conducted in the same way as many Thematic Analysis studies including one from the same research group on Severe and Enduring Anorexia Nervosa [20].

*Dependability* The study was performed over a two year period and findings throughout the study suggested that similar issues were arising from different participants.

*Confirmability* The findings from each interview were presented to the senior researcher and other members of the research group and the output of each interview was agreed in the group.

*Transferability* The findings were consistent with clinical practice and we believe that they reflect the experience of many people with longstanding BN.

*Authenticity* We believe we have explored and realistically described the lives and experiences of participants.

## Discussion

In this study we show that in a group of individuals with Bulimia Nervosa of long duration, substantial impairment is reported. Given the severity of the social, family, financial and occupational impacts of the eating disorder, we wish to contribute to a discussion on whether the term Severe and Enduring can be applied to Bulimia nervosa (SEED-BN). The proposal in relation to AN is not uncontroversial. Raykos et al [17] in a study showing that people living with long standing AN responded as well to CBT-E as other participants, question the validity of the “Severe and enduring” construct. They state *“In the absence of a meaningful construct of “severe and enduring” AN, it follows that treatment recommendations for AN should be the same, regardless of illness duration or severity.”* However, Yager [29] in a paper on harm reduction approaches in AN states *“Denial by clinicians that some patients suffer from (currently) intractable cases of SEAN* (Severe and Enduring Anorexia Nervosa*) suggests significant wilful blindness.”* Moreover, the terms used for severe and enduring eating disorders have varied between SEED [18] and SE-AN [23].

Regarding SEED-BN, a number of studies and reviews have identified a group of people suffering from BN who remain unwell, even after first line treatments [21]. The rate of “chronicity” is estimated at 22% [21]. Whereas, treatment of Severe and Enduring Anorexia Nervosa has been developed with reduced priority given to the primary symptom of AN, namely weight loss [23] no similar proposals have been made for the treatment of Severe and Enduring BN.

Although there is no agreed definition of SEED-BN we believe that the concept should not be discarded. It is clear that a proportion of people with BN have a chronic course of the illness, whatever treatment they receive [9, 21, 22]. The approach to care for people with eating disorders that have not responded to treatment is a profound challenge for clinicians. All our participants had received psychological treatment and some had found it helpful. Nevertheless all still suffered from continuing eating disorder symptoms, and some had appreciated being able to share their experiences with other people with eating disorders. The argument in favour of the SEED-BN label is that the designation draws attention to the wide range of problems faced by individuals with longstanding eating disorders. They may also afflict people with shorter illnesses, but for the individual with a long illness, the passing of years with disability add a measure of severity due to longevity. A Delphi study [6] noted importantly “*The authors acknowledge that a classification system entails a risk of stigmatizing these individuals with the potential for them to be refused the same treatment opportunities as individuals at earlier stages of the disease.”* Such stigmatization is clearly undesirable. Treasure et al [25] suggest a “*Staging model*” for eating disorders from the acute presentation to the severe and enduring stage. They considered that the model was useful for charting the course of AN but less so for BN and BED due to the lack of adequate research. Moreover, the very idea of SEED might be interpreted as conveying a hopeless prognosis. In fact Westmoreland and Mehler [28] discuss the idea of palliative care in people living with anorexia nervosa who have received many episodes of treatment that no longer feel, to the individual, beneficial. From the present study, we believe that particular attention to psychological, social and relationship problems arising from the continued eating disorder have the potential to improve quality of life even if eating disorder symptoms continue. These interventions would range from psychological and relationship therapies to medication and social and occupational interventions.

### Strengths and limitations

The present study had a small number of participants, although that is frequently the case for qualitative studies, and the number is consistent with expert advice. However, studies of long term bulimia nervosa are few, and this study being one of the early examples is a strength. The participants were drawn from a clinical service in a large city and they are likely to be representative of people with BN receiving treatment at specialist centres. Qualitative research was planned and carried out according to recognised authorities on Thematic Analysis and analysis was rigorous and closely supervised.

## Conclusions

In conclusion, 12 participants with bulimia nervosa of more than 5 years duration had highly significant difficulties in diverse realms. In treating a person with any eating disorder which has persisted for many years and in whom existing therapies have not resulted in the disorder being cured, the health worker should think broadly about the range of interventions, including self help, that might be beneficial. We believe that no treatment should be withheld on grounds of length or severity of illness if a person requests it. However, providing therapies which have failed to help in the past may not be the most creative approach, and we suggest that clinicians consider treatment which focuses on enhancing quality of life in general, rather than focussing exclusively on reduction of eating disorder symptoms. This may include social, occupational therapy, psychological, medical and dietetic approaches, while acknowledging that symptoms may remit at any time after many years, with or without treatment.

*Future directions* We recommend further research to study long term eating disorders, both qualitative and quantitative, and the development of novel approaches to their treatment. Future research should include participants views about assessment, treatment and the value or otherwise of the concept “Severe and enduring”. Moreover, the study raises questions about the proposed category of Severe and Enduring Bulimia Nervosa. How should severity be quantified? Perhaps continuing to meet research criteria for BN is an option. How should we measure enduring? This is very difficult as people with eating disorders can recover at any time, although the likelihood of recovery does reduce with time. The best we can currently do is to choose a number of years that seems long, such as 5 or 10 years, but always be aware that recovery can still occur. Broomfield et al [5] found that different authors used 5, 7 or 10 years to define SE-AN and only some required that the patient should have failed treatments. The severe and enduring term is rarely used with adolescents, so an age cutoff might be appropriate. Lastly, apart from length of illness and lack of response to treatment there is at present no reliable way to distinguish SEED from other presentations of eating disorders.

## Data Availability

No data is available for this study. In view of the private nature of the interview data, no consent was sought to make the data available beyond the researchers. No datasets were generated or analysed during the current study.
